# The Multidimensional Battery of Prosody Perception (MBOPP)

**DOI:** 10.12688/wellcomeopenres.15607.2

**Published:** 2021-10-06

**Authors:** Kyle Jasmin, Frederic Dick, Adam Taylor Tierney

**Affiliations:** 1 Department of Psychology, Royal Holloway, University of London, Ehgam, TW20 0EX, UK; 2 Psychological Sciences, Birkbeck University of London, London, WC1E 7HX, UK

**Keywords:** prosody, auditory, language, pitch, duration

## Abstract

Prosody can be defined as the rhythm and intonation patterns spanning words, phrases and sentences. Accurate perception of prosody is an important component of many aspects of language processing, such as parsing grammatical structures, recognizing words, and determining where emphasis may be placed. Prosody perception is important for language acquisition and can be impaired in language-related developmental disorders. However, existing assessments of prosodic perception suffer from some shortcomings.  These include being unsuitable for use with typically developing adults due to ceiling effects and failing to allow the investigator to distinguish the unique contributions of individual acoustic features such as pitch and temporal cues. Here we present the Multi-Dimensional Battery of Prosody Perception (MBOPP), a novel tool for the assessment of prosody perception. It consists of two subtests: Linguistic Focus, which measures the ability to hear emphasis or sentential stress, and Phrase Boundaries, which measures the ability to hear where in a compound sentence one phrase ends, and another begins. Perception of individual acoustic dimensions (Pitch and Duration) can be examined separately, and test difficulty can be precisely calibrated by the experimenter because stimuli were created using a continuous voice morph space. We present validation analyses from a sample of 59 individuals and discuss how the battery might be deployed to examine perception of prosody in various populations.

## Introduction

### Multiple dimensions for prosody

**Figure 1.**
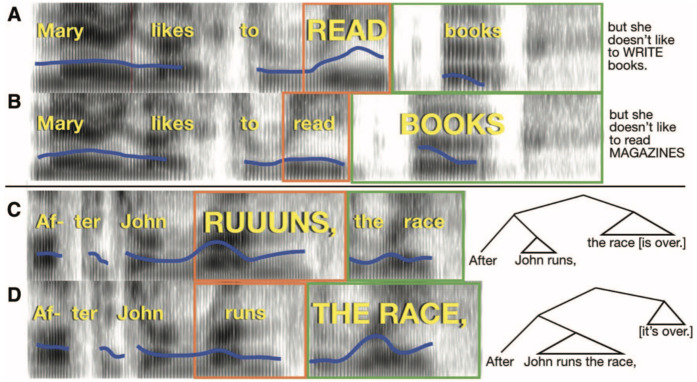
Pitch and duration (time) correlates of emphatic accents and phrase boundaries. Example spectrograms of stimuli used in the experiment (time on horizontal axis, frequency on vertical axis, and amplitude in grayscale), with linguistic features cued simultaneously by pitch and duration (the “Combined” condition). The blue line indicates the fundamental frequency of the voice. The width of the orange and green boxes indicates the duration of the words within the box. ( **A**) An emphatic accent places focus on “read”. Completion of the sentence appears to the right. ( **B**) An emphatic accent places focus on “books”; sentence completion is at right. ( **C**) A phrase boundary occurs after “runs”. ( **D**) A phrase boundary occurs after “race”. Syntactic trees are indicated at right to illustrate the structure conveyed by the acoustics of the stimuli.

One of the main tasks in speech perception is categorizing a continuous stream of speech sounds into linguistically informative phonemes or syllables ( Holt & Lotto, 2010). However, speech contains acoustic patterns on longer time scales as well. These suprasegmental or *prosodic* patterns convey crucial disambiguating lexical, syntactic, and emotional cues that help the listener capture the intended message of the talker. In English, prosodic features can be conveyed by many acoustic dimensions, including changes in pitch, amplitude, and the duration of elements. For example, prosodic focus, which helps listeners direct attention to particularly important words or phrases in a sentence, is typically cued by an increase in the amplitude and duration of the emphasized elements, along with exaggerated pitch excursion ( Breen *et al.*, 2010; Fry, 1958; see Figure 1a, b for an example). Listeners can use focus to determine the portion of the sentence to which they should be directing their attention. Similarly, lexical stress is cued by a combination of increased amplitude, pitch changes, and increased syllable duration ( Chrabaszcz *et al.*, 2014; Mattys, 2000). Listeners can use stress to help distinguish between different words (i.e. “PREsent” versus “preSENT”) and to detect word boundaries ( Nakatani & Schaffer, 1978). Finally, *phrase boundaries* tend to coincide with a change in pitch and lengthening of the syllable just prior to the boundary ( Choi *et al.*, 2005; Cumming, 2010; de Pijper & Sanderman, 1994; Streeter, 1978).

Listeners can make use of such prosodic cues to clarify potentially ambiguous syntactic structures in a sentence ( Beach, 1991; Frazier *et al.*, 2006; Jasmin *et al.*, 2020; Lehiste *et al.*, 1976; Marslen-Wilson *et al.*, 1992). In fact, prosodic patterns may be a more powerful cue to phrase structure than statistical patterns, as artificial grammar learning experiments have shown that when prosodic cues and transitional probabilities are pitted against one another, listeners will learn hierarchical structure which reflects prosodic information ( Langus *et al.*, 2012).

### Prosody and reading acquisition

Given the useful information prosodic cues provide about the structure of language, accurate prosody perception may be a crucial foundational skill for successful acquisition of language. Indeed, phonemic and prosodic awareness are independent predictors of word reading ( Clin *et al.*, 2009; Defior *et al.*, 2012; Goswami *et al.*, 2013; Holliman *et al.*, 2010a; Jiménez-Fernández *et al.*, 2015; Wade-Woolley, 2016; for a review see Wade-Woolley & Heggie, 2015), suggesting that prosody perception forms a separate dimension of linguistic skill relevant to reading acquisition. The link between prosody and reading is not limited to word reading, as prosody perception and production have also been shown to be related to reading comprehension ( Holliman *et al.*, 2014). Prosody predicts reading comprehension even when a variety of additional linguistic variables are accounted for, including phonological skills and vocabulary ( Breen *et al.*, 2016; Holliman *et al.*, 2010b; Lochrin *et al.*, 2015; Whalley & Hansen, 2006), syntactic awareness ( Veenendaal *et al.*, 2014), and decoding ( Groen *et al.*, 2019). This link between prosodic skills and reading comprehension could reflect links between prosodic and syntactic processing during reading. Fodor (1998), for example, proposed that readers generate prosodic contours during silent reading, and that these prosodic structures can affect syntactic parsing decisions, a hypothesis later supported by eye-tracking data ( Kentner, 2012).

Not only has dyslexia been linked to impaired prosody perception ( Goswami *et al.*, 2010; Holliman *et al.*, 2010a; Mundy & Carroll, 2012; Wade-Woolley, 2016; Wood & Terrell, 1998), but in adolescents with dyslexia, difficulties with the perception of lexical stress have been shown to be more prominent than problems with segmental phonology ( Anastasiou & Protopapas, 2015). Finally, prosodic sensitivity also predicts word reading one year later ( Calet *et al.*, 2015; Holliman *et al.*, 2010b), suggesting that prosody perception is a foundational skill upon which children draw when learning to read.

Such links between prosodic awareness and language acquisition suggest that the difficulties with prosody perception that accompany certain clinical diagnoses may have consequences for language acquisition. For example, some individuals with autism spectrum disorders (ASD) produce speech which lacks the usual acoustic characteristics which mark particular prosodic features; for example, the difference in duration between stressed and unstressed syllables tends to be smaller in the speech of children with ASD ( Paul *et al.*, 2008). These prosodic production deficits extend to perception as well: individuals with ASD tend to have difficulty with the perception of prosodic cues to emotion ( Globerson *et al.*, 2015; Golan *et al.*, 2007; Kleinman *et al.*, 2001; Philip *et al.*, 2010; Rutherford *et al.*, 2002), lexical stress ( Kargas *et al.*, 2016), phrase boundaries ( Diehl *et al.*, 2008), and linguistic focus ( Peppé *et al.*, 2011) in speech (but see Diehl *et al.*, 2015). These prosody perception difficulties can interfere not only with communication skill and sociability ( Paul *et al.*, 2005), but may also increase the risk of delayed language acquisition given the importance of prosody for disambiguating language meaning ( Lyons *et al.*, 2014).

### Prosody and language disorders

Prosody perception is, therefore, a vital skill supporting language development, and is impaired in several clinical populations in which there is intense interest. As mentioned above, prosodic features tend to be conveyed by a mixture of multiple different cues, including changes in the pitch and duration of syllables and words. As a result, one source of difficulties with prosody perception may be impairments in auditory processing, a possibility supported by findings that prosody perception in children correlates with psychophysical thresholds for pitch, duration, and amplitude rise time ( Goswami *et al.*, 2013; Haake *et al.*, 2013; Richards & Goswami, 2015). However, impairments in auditory processing can be present for one dimension in the presence of preserved processing in other dimensions. In particular, impaired pitch perception can co-occur with preserved duration perception (and vice versa - Kidd *et al.*, 2007). Similarly, research on amusia has shown that highly impaired memory for pitch sequences can co-occur with preserved memory for durational sequences ( Hyde & Peretz, 2004). A prosody perception deficit in a given individual, therefore, could reflect impaired pitch perception or duration perception or both. Existing methodologies for assessing prosody perception, however, cannot control the acoustic cues to different prosodic features, and therefore cannot diagnose the source of an individual’s prosodic impairment.

### Existing prosody tests

Although there exist many widely available standardized tests of segmental speech perception usable by individuals of all ages ( Killion *et al.*, 2004; Nilsson *et al.*, 1994; Wilson, 2003), there are comparatively few instruments publicly available for researchers and clinicians interested in testing suprasegmental speech perception. Consequently, prosody perception research has been carried out using a wide variety of in-house methods developed within single laboratories, making comparison across studies difficult. These include perceptual matching tasks such as matching low-pass filtered sentences or indicating whether the prosodic structure of low-pass filtered sentences match unfiltered target sentences ( Cumming *et al.*, 2015; Fisher *et al.*, 2007; Wood & Terrell, 1998). Participants have also been asked to match the stress pattern of a nonsense phrase like “DEEdee DEEdee” with a spoken target phrase like “Harry Potter” ( Goswami *et al.*, 2010; Holliman *et al.*, 2012; Mundy & Carroll, 2012; Whalley & Hansen, 2006). These tests have the advantage of isolating the suprasegmental elements of speech. However, because these tests do not use actual language, they arguably measure auditory discrimination rather than prosody perception per se. Moreover, these tests are not publicly available.

A widely used battery of prosody perception available for purchase by the public is the Profiling Elements of Prosodic Systems—Children test, or PEPS-C ( Peppé & McCann, 2003). This test assesses the perception and production of four different aspects of prosody: affect, phrase structure, focus, and interaction. Each subtest features two different sets of trials. In “form” trials, the listener is asked to make same/different judgments on utterances which either do or do not differ based on a prosodic feature. In “function” trials, the listener is asked to infer the speaker’s intent by detecting a prosodic feature. For example, one item from the phrase structure subtest asks listeners to point to the picture that best fits the utterance “fish, fingers, and fruit” (as opposed to “fish fingers and fruit”; NB:British English “fish fingers” are called “fish sticks” in American English). This test has been successfully used to study a variety of topics related to prosody perception in children, including the relationship between prosody perception and reading ability in typically developing children ( Lochrin *et al.*, 2015), and impairments in prosody perception in children with specific language impairment, dyslexia, and ASD ( Jarvinen-Pasley *et al.*, 2008a; Marshall *et al.*, 2009; Wells & Peppé, 2003).

The main limitation of the PEPS-C is that it was designed to be administered to children, and therefore many adults would perform at ceiling. The PEPS-C was adapted from an earlier battery designed to be used with adults (the PEPS), but it is not available for use by the public, and there is also evidence for the existence of ceiling effects in adult PEPS data ( Peppé *et al.*, 2000). Moreover, there are a number of examples of ceiling effects in the literature on prosody perception in adolescents and adults in research using other prosody perception tests ( Chevallier *et al.*, 2009; Lyons *et al.*, 2014; Paul *et al.*, 2005), suggesting that existing methodologies for testing prosody perception are insufficiently challenging for adult participants. Research on prosody would be facilitated by a publicly available test with adaptive difficulty suitable for a range of ages and backgrounds.

### The current study

Here we report and make publicly available the Multidimensional Battery of Prosody Perception (MBOPP), a battery of prosody perception with adaptive difficulty which is therefore suitable for participants of all ages, backgrounds, and ability levels. This battery consists of two tests, one assessing the perception of linguistic focus and another assessing the perception of phrase boundaries. For both tests, stimuli were constructed by asking an actor to read aloud sequences of words which were identical lexically but differed on the presence of a prosodic feature. Thus, each sentence in the focus test has an “early focus” and “late focus” version, referring to the relative position of emphasized elements. Similarly, the sentences in the phrase test have an “early closure” and “late closure” version, referring to the placement of the phrase boundary (indicated typographically with a comma). Speech morphing software (STRAIGHT, Kawahara & Irino, 2005) was then used to decompose these two recordings, align them onto one another, and resynthesize (“morph”) them such that the extent to which pitch and durational patterns cued one prosodic interpretation or the other could be varied independently while all other acoustic characteristics are set to be intermediate between the two recordings. This method allows the researcher to tune the difficulty of the test to any population (by choosing which subset of stimuli to use) and enables investigation of dimension-specific prosody perception. This test was presented to 57 typically developed adult participants to examine the relative usefulness of pitch versus durational cues for focus and phrase boundary perception, and to measure the reliability of each subtest.

## Methods

### Participants

Participants (N=59, 34F, 24M, 1 non-binary by self-ID, aged 29.0±6.1) were recruited using Prolific – an online participant recruitment portal – in exchange for payment after the session. All participants were native English speakers of British English. The same participants completed both the focus perception and phrase perception tasks.

### Materials – Focus Perception

The Focus Perception test consists of 47 compound sentences (two independent clauses separated by a conjunction; Table 1). We recorded spoken versions of these sentences in a quiet room using a Rode NT1-A condenser microphone (44.1 kHz, 32-bit) as they were spoken by a former professional actor, now a speech researcher. The actor placed contrastive accents to emphasize the capitalized words in the sentences. Each of the sentences was read with emphasis on two different word pairs, thus creating two versions: an “early focus” version (e.g., “ Mary likes to READ books, but she doesn’t like to WRITE them,” focus indicated by upper-case letters), and “late focus”, where the focus elements occurred in later positions in the sentence (e.g., “ Mary likes to read BOOKS, but she doesn’t like to read MAGAZINES,” focus indicated by upper-case letters; Figure 1a, b). Thus, the emphasis placed on the words in capitalized letters served to indicate contrastive focus, meant to indicate which linguistic elements (words, in this case) should receive greater attention to clarify the speaker’s intentions. For example, suppose the conversation began as follows:

A. Why doesn’t Mary like books?
B. She likes to READ books, but not WRITE them.

**Table 1.** Text of Focus Stimuli Sentences.

#	Start	Focused Word 1	Focused Word 2	Middle	Ending 1	Ending 2
**1**	Mary likes to	read	books	but she doesn’t like to	WRITE books	read MAGAZINES
**2**	Alice sometimes	pets	dogs	but she won’t	WASH dogs	pet CATS
**5**	Dave likes to	study	music	but he doesn’t like to	PLAY music	study HISTORY
**6**	Sally has a	Windows	computer	but she really wants	an APPLE computer	a Windows TABLET
**7**	George asked for a	white	Americano	but the barista gave him a	BLACK Americano	white filter COFFEE
**8**	Fiona was eating	strawberry	yoghurt	but she really wanted some	BLUEBERRY yoghurt	strawberry ICECREAM
**9**	Tom likes	barbecue	chicken	but not as much as	ROAST chicken	barbecue PORK
**10**	Sophie likes to	paint	landscapes	but she doesn’t like to	DRAW landscapes	paint PORTRAITS
**11**	John can’t	run	a marathon	but he could	WALK a marathon	run a MILE
**12**	Matt is good at	flying	planes	but he isn’t good at	LANDING planes	flying HELICOPTERS
**13**	Pippa found a	jam	jar	but she couldn’t find a	JELLY jar	jam KNIFE
**14**	Sam has a	fish	knife	but he doesn’t have a	BUTTER knife	fish FORK
**15**	Rachel likes	French	food	but she doesn’t like	ITALIAN food	French WINE
**16**	The woman likes	white	pearls	but not	BLACK pearls	white DIAMONDS
**17**	Ken won’t buy	Sainsbury’s	pizza	but he will buy	TESCO’S pizza	Sainsbury’s CHICKEN
**18**	Sarah has a	Barclay’s	card	but she doesn’t have a	LLOYDS card	Barclay’s MORTGAGE
**19**	Neil won’t support	Oxford’s	fencing team	but he will support	CAMBRIDGE’S fencing team	Oxford’s ROWING team
**20**	Carolyn likes	Scottish	pubs	but she doesn’t like	ENGLISH pubs	Scottish RESTAURANTS
**21**	Micah has been to	Regent’s	park	but he hasn’t been to	HYDE Park	Regent’s STREET
**22**	Rosalyn likes to	drink	beer	but she doesn’t like to	BREW beer	drink LIQUOR
**23**	Veronica has visited	America	for holiday	but she hasn’t visited	CANADA for holiday	America FOR WORK
**24**	Tim has an	electric	piano	but he really wants an	ACOUSTIC piano	electric GUITAR
**25**	Ben has ridden a	UK	train	but he has never ridden a	AMERICAN train	UK BUS
**26**	Nancy has a	small	flat	but she would really like a	LARGE flat	small HOUSE
**27**	Paul’s house has a	brown	sofa	but it doesn’t have a	BLACK sofa	brown CHAIR
**28**	Robert doesn’t like	Dutch	cinema	but he does like	GERMAN cinema	Dutch THEATRE
**29**	Jenny doesn’t have any	ginger	friends	but she does have several	BLONDE friends	ginger COLLEAGUES
**30**	You shouldn’t open the	red	suitcase	but you can open the	GREEN suitcase	red CHEST
**31**	Emma doesn’t	speak	well	but she does	DRESS well	speak OFTEN
**32**	Rose has visited	southern	Greece	but she has not visited	NORTHERN Greece	southern ITALY
**33**	Jane can speak	modern	Greek	but she can’t speak	ANCIENT Greek	modern EGYPTIAN
**34**	Jim likes	Boots’	shampoo	but he doesn’t like	SUPERDRUG shampoo	Boots’ BODYWASH
**35**	Cameron will sometimes	watch	basketball	but he will never	PLAY basketball	watch CRICKET
**36**	Terry buys	sparkling	water	but not	STILL water	sparkling WINE
**37**	Richard said to buy	red	cups	but not	BLUE cups	red PLATES
**38**	Harriet can	speak	Mandarin	but she can’t	READ Mandarin	speak CANTONESE
**39**	Olivia was looking for	wooden	boats	but she only found	PLASTIC boats	wooden PLANES
**40**	Michael likes to	plant	flowers	but he hates to	PICK flowers	plant POTATOES
**41**	Cathy likes to	observe	children	but she doesn’t like to	TALK to children	observe ADULTS
**42**	Lily likes to	buy	stocks	but she doesn’t like to	SELL stocks	buy BONDS
**43**	Alex likes to	collect	dolls	but he doesn’t like to	PLAY with dolls	collect STAMPS
**44**	Frank has a	toy	dog	but he would really like a	REAL dog	toy BIRD
**46**	Bonnie has an	American	visa	but she really wants a	BRITISH visa	American PASSPORT
**47**	Patsy likes	Starbucks	coffee	but her friends like	COSTA coffee	Starbucks TEA
**48**	Timothy bought a	leather	jacket	because he couldn’t find	a CLOTH jacket	leather SHOES
**49**	Carrie likes	Star Trek	films	but she can’t stand	Star WARS films	Star TREK cartoons
**50**	Daniel enjoys	Chicago	pizza	but he doesn’t care for	NEW YORK pizza	Chicago BEER

The focused elements spoken by B serve to contrast with the presupposition by speaker A. The terms “early focus” and “late focus” used in this article refer simply to which pair of words is emphasized (e.g. READ and WRITE occur earlier than BOOKS and MAGAZINES, respectively.)

The audio recordings of these sentences were trimmed such that they included only the first clause, which consisted of identical words in each version (this clause is indicated in the examples above via underlining). The raw recordings of “early” and “late” focus sentences were then morphed together to create intermediate versions. Morphing was performed with STRAIGHT software ( Kawahara & Irino, 2005). The two recordings of each sentence (differing only in the placement of the emphasized word) were manually time-aligned by examining a similarity matrix created from the two recordings and manually marking anchor points at energy changes (e.g. bursts) in each recording. After establishing these anchor points, morphed intermediate versions of the sentences were synthesized. An experimenter listened to the result of the morphing to check the quality of the output. If quality was low, anchor points were added or adjusted and the procedure was repeated until the resulting morph sounded natural. STRAIGHT allows morphs along several dimensions: Aperiodicity, Spectrum, Frequency, Time (duration), and F0 (pitch). For the morphs created for this prosody battery, only Duration and Pitch were manipulated.

We are distributing this stimulus set (see *Extended data*; Jasmin, 2021) with morphs in three conditions: Pitch, Time, and Combined. The Combined condition consists of stimuli in which duration and pitch information cue emphasis on the same word -- either early focus or late focus (e.g. Mary likes to READ books vs Mary likes to read BOOKS). Morphing rates are expressed in terms of percent, such that lower values indicate more information from the early focus recording, and higher values indicate more information from the late focus recording, while 50% indicates an equal amount of a given dimension from each recording.

For stimuli in the Pitch condition, the emphasized word in the sentence is conveyed by pitch cues alone which vary from 0% (pitch information coming entirely from the early focus recording) to 100% (pitch information coming from the late focus recording), while duration cues are ambiguous with the Time parameter always set at 50%. In the Duration condition, emphasis is conveyed only by durational cues, which similarly vary from 0% to 100%, while pitch cues are ambiguous, always set at 50%. The other morphing dimensions available in STRAIGHT (Aperiodicity, Spectrum, and Frequency) were held at 50% such that morphs contained equal amounts of information from the two recordings.

Table 2 displays the morphing rates included in the stimuli published with this article. The file naming format for the stimuli is as follows.

[Stimulus number] _ [pitch morphing rate] _ [duration morphing rate] .wav

Examples:

• Focus1_pitch0_time0.wav – pitch and duration both cue EARLY focus (Combined)
• Focus1_Pitch100_time100.wav – pitch and duration both cue LATE focus (Combined)
• Focus1_pitch50_time0.wav – pitch is ambiguous, only duration cues EARLY focus (Time)
• Focus1_pitch50_time100.wav – pitch is ambiguous, only duration cues LATE focus (Time)
• Focus1_pitch0_time50.wav – duration is ambiguous, only pitch cues EARLY focus (Pitch)
• Focus1_pitch100_time50.wav – duration is ambiguous, only pitch cues LATE focus (Pitch)

**Table 2.** Morphing rates for Phrase and Focus test stimuli.

Condition	Pitch Morphing Rate	Duration Morphing Rate
Pitch	0% to 40%, 60 to 100%, in 5% increments	Always 50%
Duration	Always 50%	0% to 40%, 60 to 100%, in 5% increments
Combined	0% to 40%, 60 to 100%, in 5% increments	0% to 40%, 60 to 100%, in 5% increments

For the experiments included in this report, these six different kinds of morphs were created by varying the amount of pitch-related and time information either independently or simultaneously. For the Pitch condition, duration morphing rates were held at 50%, while two contrasting pitch versions were created at 25% (towards early focus) and 75% (towards late focus). For the Duration condition, pitch was held at 50% while duration was manipulated to be 25% (early focus) or 75% (late focus). For the Combined condition, both the pitch and the Duration dimensions were manipulated simultaneously to be 25% or 75%. Morphing rates of 25% (instead of 0%) and 75% (instead of 100%) were used to make the task more difficult. The task could be made yet more difficult by moving these values even closer to 50% (e.g. 40% for early focus and 60% for late focus). All files were saved and subsequently presented at a sampling rate 44.1 kHz with 16-bit quantization.

For the experiments included in this report, these six different kinds of morphs were created by varying the amount of pitch-related and time information either independently or simultaneously. For the Pitch condition, duration morphing rates were held at 50%, while two contrasting pitch versions were created at 25% (towards early focus) and 75% (towards late focus). For the Duration condition, pitch was held at 50% while duration was manipulated to be 25% (early focus) or 75% (late focus). For the Combined condition, both the pitch and the Duration dimensions were manipulated simultaneously to be 25% or 75%. Morphing rates of 25% (instead of 0%) and 75% (instead of 100%) were used to make the task more difficult. The task could be made yet more difficult by moving these values even closer to 50% (e.g. 40% for early focus and 60% for late focus). All files were saved and subsequently presented at a sampling rate 44.1 kHz with 16-bit quantization.

The text of the stimuli are given in Table 1. The auditory recordings consist of the following portions of the text: Start, Focused Word 1, Focused Word 2.

### Procedure – Focus Perception

Performance and reliability data reported here were collected with Gorilla Experiment Builder ( Anwyl-Irvine *et al.*, 2019). We tested participants’ ability to detect prosodic differences by asking them to match auditory versions of sentences with text ones. Participants read sentences presented visually on the screen one at a time, which were either early or late focus. For example, one visually presented sentence was “Mary likes to READ books, but she doesn’t like to WRITE books.”

The emphasized words appeared in all upper-case letters, as in the example above. Subjects were then given 4 seconds to read the sentence to themselves silently and imagine how it should sound if someone spoke it aloud. Following this, subjects heard the early focus and late focus versions of the first independent clause of the stimulus sentence (up to but not including the conjunction). The order of the presentation was randomized. Participants decided which of the two readings contained emphasis placed on the same word as in the text sentence and responded by pressing “1” or “2” on the keyboard to indicate if they thought the first version or second version was spoken in a way that better matched the on-screen version of the sentence. The stimuli were divided into three lists (47 trials each) and counterbalanced such that participants heard an equal number of Pitch, Duration and Combined stimulus examples. For 23 of the stimuli, presentations featured the early focus version; for the remaining stimuli, the presentation was late focus. Each participant judged each stimulus in each of the conditions, spread across the 3 lists. The entire task lasted approximately 30 minutes.

### Materials – Phrase Perception

The Phrase Perception test stimuli consisted of 42 pairs of short sentences with a subordinate clause appearing before a main clause (see Figure 1c, d). About half of these came from a published study ( Kjelgaard & Speer, 1999) and the rest were created for this test (see Table 3). The sentence pairs consisted of two similar sentences, the first several words of which were identical. In the first type of sentence, “early closure”, the subordinate clause’s verb was used intransitively, and the following noun was the subject of a new clause (“After John runs, the race is over”). In the second type of sentence, “late closure”, the verb was used transitively and took the immediately following noun as its object, which caused a phrase boundary to occur slightly later in the sentence than in the early close version (“After John runs the race, it’s over”). Both versions of the sentence were lexically identical from the start of the sentence until the end of the second noun. The same actor recorded early and late closure versions of the sentences in his own standard Southern English dialect. The recordings were cropped such that only the lexically identical portions of the two versions remained, and silent pauses after phrase breaks were removed.

**Table 3.** Text of the Phrase Test sentences, each of which has two versions, where a phrase boundary occurs either earlier or later in the sentence.

#	Closure	Start	Finish
**1**	Early	After Jane dusts, the dining table	is clean
**1**	Late	After Jane dusts the dining table,	it’s clean
**2**	Early	After John runs, the race	is over
**2**	Late	After John runs the race,	it’s over
**5**	Early	Because Mike phoned, his mother	was relieved
**5**	Late	Because Mike phoned his mother,	she was relieved
**7**	Early	Because Sarah answered, the teacher	was proud
**7**	Late	Because Sarah answered the teacher,	she was proud
**8**	Early	Because Tara cleaned, the house	was spotless
**8**	Late	Because Tara cleaned the house,	it was spotless
**9**	Early	Because George forgot, the party	had started
**9**	Late	Because George forgot the party,	he was sad
**10**	Early	Because Mike paid, the bill	was smaller
**10**	Late	Because Mike paid the bill,	it was smaller
**13**	Early	If Charles is baby-sitting, the children	are happy
**13**	Late	If Charles is baby-sitting the children,	they’re happy
**14**	Early	If George is programming, the computer	is busy
**14**	Late	If George is programming the computer,	it’s busy
**15**	Early	If Ian doesn’t notice, Beth	is fine
**15**	Late	If Ian doesn’t notice Beth,	it’s fine
**16**	Early	If Joe starts, the meeting	will be long
**16**	Late	If Joe starts the meeting,	it’ll be long
**18**	Early	If Laura is folding, the towels	will be neat
**18**	Late	If Laura is folding the towels,	they’ll be neat
**19**	Early	When the baby finishes, the bottle	will be empty
**19**	Late	When the baby finishes the bottle,	it’ll be empty
**20**	Early	If Barbara gives up, the ship	will be plundered
**20**	Late	If Barbara gives up the ship,	it’ll be plundered
**21**	Early	If the Scissor Sisters open, the show	will be great
**21**	Late	If the Scissor Sisters open the show,	it’ll be great
**22**	Early	If the maid packs, the suitcase	will be tidy
**22**	Late	If the maid packs the suitcase,	it’ll be tidy
**23**	Early	If Tom wins, the contest	is over
**23**	Late	If Tom wins the contest,	it’s over
**24**	Early	If the doctor calls, your sister	will answer
**24**	Late	If the doctor calls your sister,	she’ll answer
**25**	Early	If Jack cleans, the kitchen	will be filthy
**25**	Late	If Jack cleans the kitchen,	it’ll be filthy
**26**	Early	If dad digs, the hole	will be deep
**26**	Late	If dad digs the hole,	it’ll be deep
**27**	Early	When a man cheats, his friends	get angry
**27**	Late	When a man cheats his friends,	they’re angry
**29**	Early	When Gaga sings, the song	is a hit
**29**	Late	When Gaga sings the song,	it’s a hit
**30**	Early	When Roger leaves, the house	is dark
**30**	Late	When Roger leaves the house,	it’s dark
**31**	Early	When Suzie visits, her grandpa	is happy
**31**	Late	When Suzie visits her grandpa,	he’s happy
**32**	Early	When the clock strikes, the hour	has started
**32**	Late	When the clock strikes the hour,	it’s started
**33**	Early	When the guerrillas fight, the battle	has begun
**33**	Late	When the guerrillas fight the battle,	it’s begun
**34**	Early	When the maid cleans, the rooms	are organized
**34**	Late	When the maid cleans the rooms,	they’re organized
**35**	Early	When the original cast performs, the play	is fantastic
**35**	Late	When the original cast performs the play,	it’s fantastic
**36**	Early	When Tim is presenting, the lectures	are interesting
**36**	Late	When Tim is presenting the lectures,	they’re interesting
**37**	Early	When The Beatles play, the music	is noisy
**37**	Late	When The Beatles play the music,	it’s noisy
**38**	Early	When Paul drinks, the rum	disappears
**38**	Late	When Paul drinks the rum,	it disappears
**39**	Early	When Mary helps, the homeless	are grateful
**39**	Late	When Mary helps the homeless,	they’re grateful
**40**	Early	When the phone loads, the app	crashes
**40**	Late	When the phone loads the app,	it crashes
**41**	Early	When the shop closes, its doors	are locked
**41**	Late	When the shop closes its doors,	they’re locked
**42**	Early	When a train passes, the station	shakes
**42**	Late	When a train passes the station,	it shakes
**43**	Early	When the actor practices, the monologue	is excellent
**43**	Late	When the actor practices the monologue,	it’s excellent
**44**	Early	When the cowboy rides, the horse	is tired
**44**	Late	When the cowboy rides the horse,	it’s tired
**46**	Early	Whenever the guard checks, the door	is locked
**46**	Late	Whenever the guard checks the door,	it’s locked
**47**	Early	Whenever Bill teaches, the course	is boring
**47**	Late	Whenever Bill teaches the course,	it’s boring
**48**	Early	Whenever a customer tips, the waiter	is pleased
**48**	Late	Whenever a customer tips the waiter,	he’s pleased
**49**	Early	Whenever Rachel leads, the discussion	is exciting
**49**	Late	Whenever Rachel leads the discussion,	it’s exciting
**50**	Early	Whenever Mary writes, the paper	is excellent
**50**	Late	Whenever Mary writes the paper,	it’s excellent

Auditory stimuli for the phrase test were created in the same way as in the focus test, by asking an actor to read aloud the two versions of each sentence (the early and late closure). Then the recordings were cropped to the lexically identical portions, corresponding anchor points were defined, and morphs were created in STRAIGHT. The morphs we publish here were created with the same proportions as in the focus test ( Table 2).

***Phrase Perception test procedure.*** For the validation experiments reported here, we used stimuli with early or late closure cued by 75% and 25% morphing rates. The procedure for the Linguistic Phrase test was similar to that of the Linguistic Focus Test. On each trial, participants read a text version of each sentence online, which was either early or late closure, as indicated by the grammar of the sentence and a comma placed after the first clause ( Figure 1c, d). Participants read the sentence to themselves silently and imagined how it should sound if someone spoke it aloud. Following this, subjects heard the first part of the sentence (which was lexically identical in the early and late closure versions) spoken aloud, in two different ways, one that cued an early closure reading and another that cued a late closure reading. Participants decided which of the two readings best reflected the text sentence (and the location of its phrase boundary, indicated grammatically and orthographically with a comma) and responded by pressing “1” or “2” on the keyboard to indicate if they thought the first version or second version was spoken in a way that better matched the on-screen version of the sentence. The grammatical difference between the two spoken utterances on each trial was cued by pitch differences (Pitch), duration differences (Duration), or both pitch and duration differences (Combined). Subjects completed three blocks of 42 trials. Stimuli were counterbalanced, with half of the presentations indicating early closure and half late closure. Each participant judged each stimulus in every condition, across the 3 lists. The task was performed online using Gorilla Experiment Builder and lasted approximately 25 minutes.

### Statistical analysis

All statistical analyses were performed with R ( R Core Team, 2021). Mixed effects models were performed with the *lme4* function.

An earlier version of this article can be found on bioRxiv (DOI: https://doi.org/10.1101/555102).

## Results

### Overall performance

**Figure 2.**
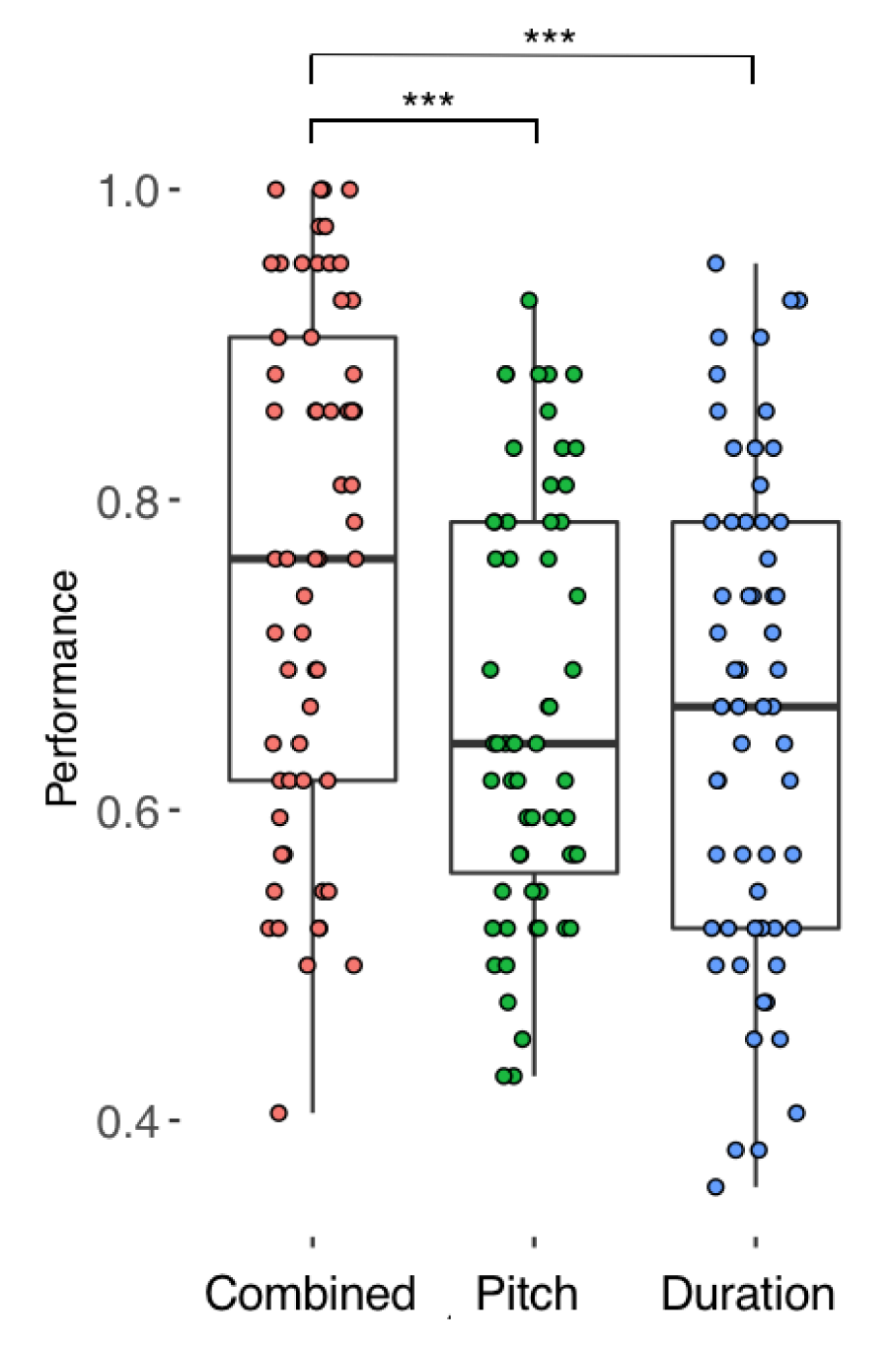
Performance across all 59 participants in each condition of the Phrase Perception test. Horizontal lines indicate median performance.

**Figure 3.**
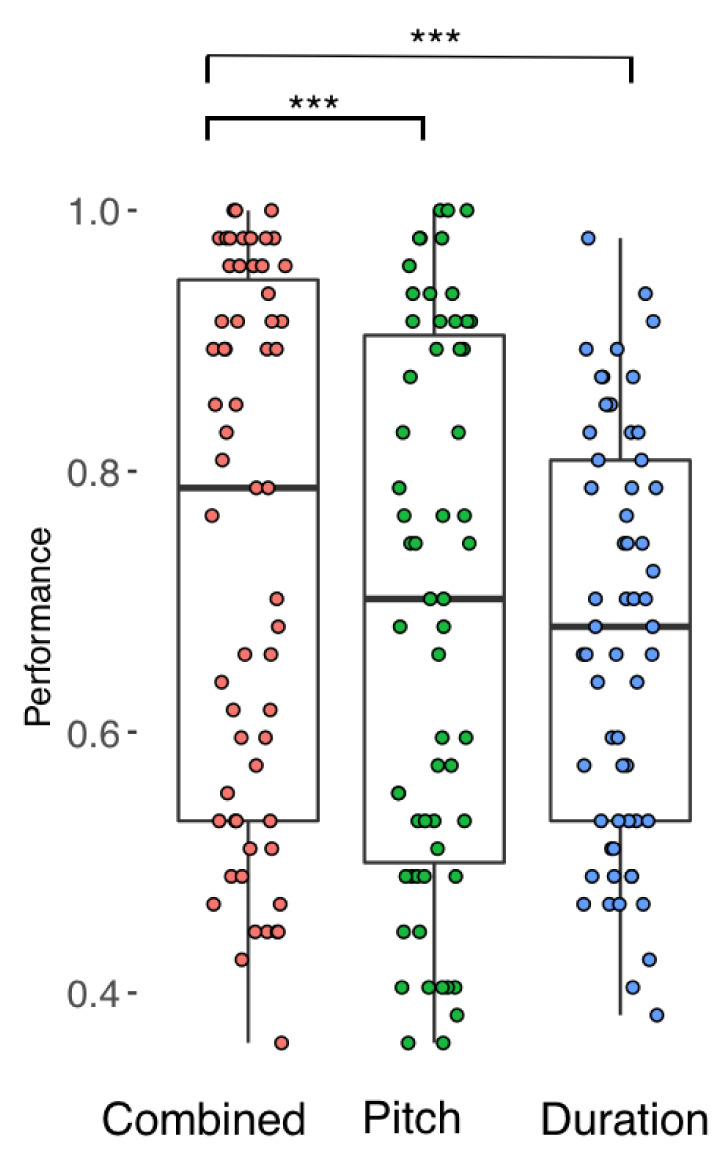
Performance across all 59 participants in each condition of the Focus Perception test. Horizontal lines indicate median performance.

Figure 2 and Figure 3 display all participants’ performance in the phrase perception and focus perception tests, respectively. Overall, there was a wide range in performance, with no evidence of ceiling or floor effects. Results from each participant are given as *Underlying data* ( Jasmin, 2021).

### Subtest reliability

Cronbach’s alpha was used to calculate reliability for each of the six subtests by first (for each condition and test) creating a matrix with a row for each subject, a column for each item, and the performance score (1 vs 0) as the value, and then submitting this matrix to the alpha function in R’s *psych* package ( Revelle, 2016). For the focus tests, reliability was 0.92 for the Pitch condition, 0.83 for the Duration condition, and 0.92 for the Combined condition. For the phrase test, reliability was 0.73 for the Pitch condition, 0.81 for the Duration condition, and 0.87 for the Combined condition. To summarize, reliability tended to be highest for the Combined condition, and reliability was somewhat higher for the focus tests than for the phrase tests. Overall, however, these reliability scores compare favorably with those of other batteries of prosody perception ( Kalathottukaren *et al.*, 2015).

### Comparison between conditions

To examine the relative usefulness of pitch and duration cues in the perception of phrase boundaries and linguistic focus we used mixed effects logistic regression with test (phrase versus focus) and condition (Combined, Pitch, and Duration) as fixed factors, and item and participant as random intercepts. Main effects of condition and task were tested by comparing the full model (Condition + Test + Condition * Test) with a null model that omitted the factor of interest and the interaction term. There was no statistically significant main effect of test (p = .06). However, there was a main effect of condition (χ ^2^(4) = 126.12, p < 0.001) and an interaction between test and condition (χ ^2^(2) = 6.92, p = 0.03).

FDR-corrected post-hoc paired t-tests revealed that for focus perception, participants performed better on the Combined condition compared to the Duration condition (OR = 1.47, Z = 6.17, p < .001) and also compared to the Pitch condition (OR = 1.34, Z = 4.63, p < .001). Performance on the Pitch and Duration conditions did not differ (OR = 1.1, Z = 1.56, p = .36). Similarly, for phrase perception, participants performed better on the Combined condition compared to the Pitch (OR = 1.70, Z = 7.96, p < .001) and Duration (OR = 1.71, Z = 8.02, p < .001) conditions. Performance on the Duration condition did not differ from the Pitch condition (OR = 1.00, Z = 0.06, p = 1). These results suggest that, across both focus and phrase perception, the presence of an additional cue was generally useful to listeners. Finally, comparisons within each condition, between the two tests was compared. Performance did not differ between the Phrase and Focus tests for the Combined condition (OR = 0.9, Z = -1.22, p = 0.22) or Duration condition (OR = 1.05, Z = 0.71, p = .48), but performance was marginally (though not significantly) higher in the Pitch condition on the Focus test (OR = 1.16, Z = 1.92, p = .055).

### Relationships between conditions

**Figure 4.**
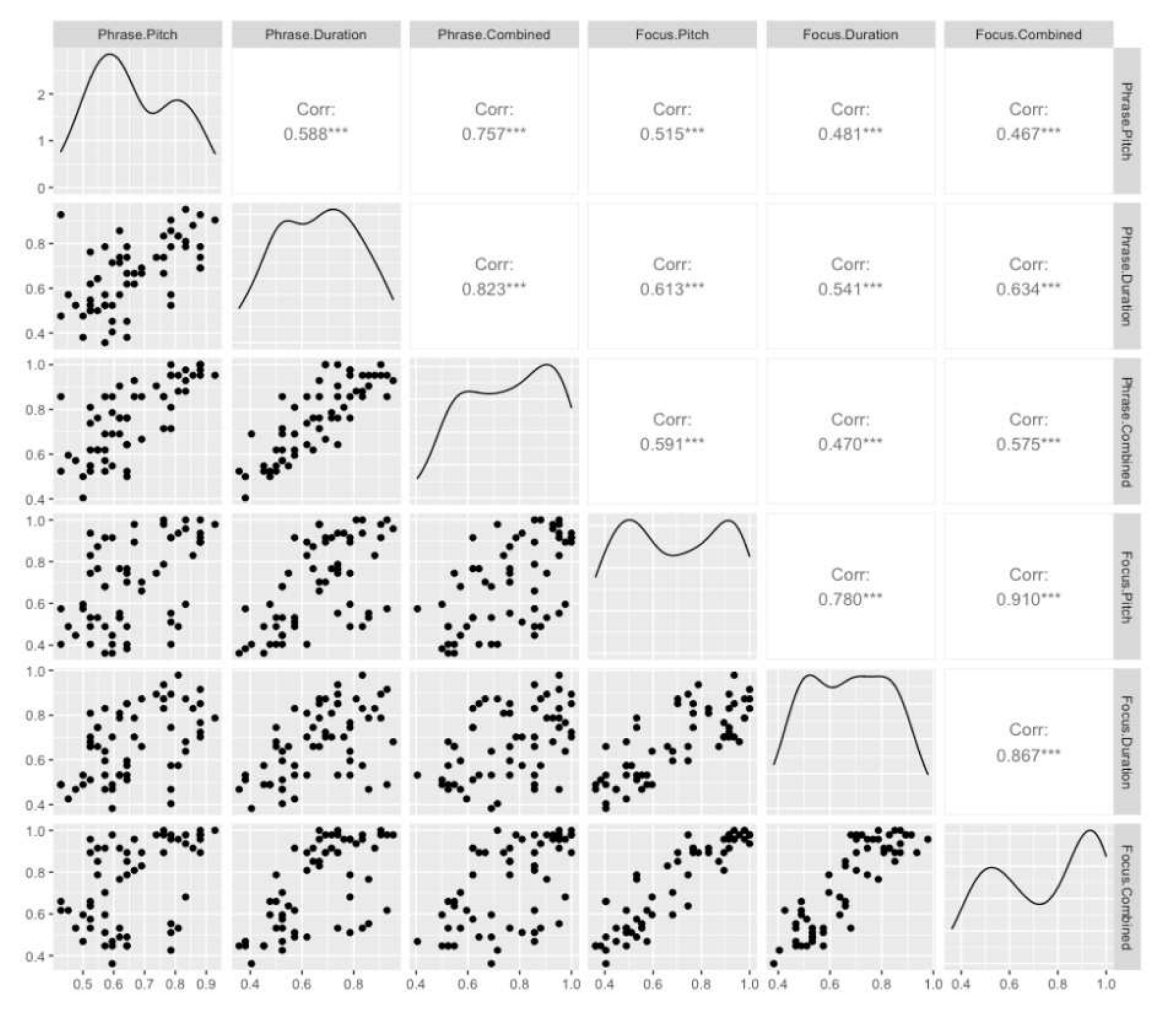
Scatterplots displaying the relationship between performance across each possible pair of all six conditions. The upper triangle shows Pearson correlation coefficients. *** indicates p<.001. The diagonal shows variable distributions.

Pearson’s correlations were used to examine the relationship between performance (proportion correct response for each subject) across all six subtests. Correlations are shown along with relationships between all six variables displayed in scatterplots, in Figure 4. Correlations between all conditions were significant, but varied in strength. Generally, correlations between subtests within each prosody test were stronger than correlations between prosody tests. For example, the correlation between performance in the Pitch condition and Duration condition of the focus perception test was r = 0.78, while the correlation between performance in the Pitch condition of the phrase test and the Duration condition of the focus perception test was r = 0.48.

The correlation data do not indicate that subtests requiring analysis of similar perceptual cues correlate more strongly. For example, the correlation between the two Duration conditions is not stronger than the correlation between the Duration condition of the focus test and the Pitch condition of the phrase test. This result raises the question of whether the Pitch and Duration conditions are, indeed, indexing different aspects of prosody perception. We investigated this question by conducting two mixed effects multiple logistic regressions, one for Focus and another for Phrase, with performance on the Combined condition (Correct vs Incorrect) as the dependent variable, and performance on the Pitch and Duration conditions (Correct vs Incorrect) as fixed effects, and Item as random effect. For focus perception, we found that Pitch performance (OR = 3.79, Z= 14.0, p < 0.001) and Duration performance (OR = 2.01, Z = 7.33, p < 0.001) explained independent variance in performance in the Combined cues condition. This suggests that perception of focus draws on both pitch and duration perception, but that pitch is relatively more important. For phrase perception, we also found that Pitch performance (OR = 1.91, Z = 6.57, p < 0.001) and Duration performance (OR = 1.62, Z = 4.91, p < 0.001) explained independent variance in performance in the Combined cues condition. This suggests that perception of phrase boundaries draws on both pitch and duration perception, and that both cues are relatively equally important.

## Discussion

Here we have presented a new battery of prosody perception which is suitable for examining prosody perception in adults. This instrument could facilitate investigation of a number of research questions, such as whether difficulties with prosody perception in individuals with dyslexia or ASD extend into adulthood. Another avenue of investigation would be dialectal variation (see Fuchs, 2016), e.g. whether speakers of other varieties of English are able to use pitch and duration similarly. Second language learning may also be a fruitful line of research using the battery. Indeed, we have recently shown that L2 English speakers of L1 Mandarin tend to perceptually weight pitch highly in perception of English speech ( Jasmin *et al.*, 2021). This battery could also be used to test the hypothesis that musical training can enhance focus and phrase boundary perception. This possibility is supported by findings that musical training is linked to enhanced encoding of the pitch of speech ( Bidelman *et al.*, 2011; Marques *et al.*, 2007; Moreno & Besson, 2005; Musacchia *et al.*, 2007; Wong *et al.*, 2007) and syllable durations ( Chobert *et al.*, 2011) and that musicians are better than non-musicians at detecting stress contrasts ( Kolinsky *et al.*, 2009) and discriminating statements from questions based on intonational contours ( Zioga *et al.*, 2016).

### Adaptive difficulty

The test stimuli for the MBOPP were created using speech morphing software. As a result, the test difficulty is fully customizable (because researchers can select the stimuli with desired cue magnitude) without compromising ecological validity and natural characteristics of the stimuli. The data reported here were collected by setting prosodic cue size to medium levels. This resulted in data that largely avoided both floor and ceiling effects in typically developing adults, although there was some evidence of ceiling performance in the Pitch and Combined cues conditions of the focus perception test. This suggests that to equate difficulty across the focus and phrase perception tests the cue size for the focus perception test should be slightly lower than that for the phrase perception test.

Given that cue size was set here at 50% of maximum, there remains quite a bit of scope for lowering the difficulty of the test to make it appropriate for other populations who may have lower prosody perception skills, such as children or adults with perceptual difficulties. The ability to modify cue size on a fine-grained level also enables researchers to modify test difficulty on an item-by-item basis. This could have two important uses. First, adaptive prosody perception tests could allow researchers to rapidly find participants’ thresholds for accurate prosody perception by modifying test difficulty in response to participants’ performance, enabling the use of shorter test protocols. And second, adaptive prosody perception training paradigms could be created by ensuring that participants are presented with stimuli at a difficulty level that is neither so easy as to be trivial nor so difficult as to be frustrating.

### Independent modification of individual cues

Another novel feature of the MBOPP is the ability to modify the size of pitch and duration cues independently. This makes possible investigations into whether prosody perception deficits are dimension-specific in certain populations. For example, we have demonstrated using the MBOPP that adults with amusia demonstrate impaired focus perception in the Pitch condition but perform similarly to typically developing adults on the Duration condition ( Jasmin *et al.*, 2020). Investigating the dimension specificity of prosody perception deficits is one way to test the hypothesis that difficulties with prosody perception in a given population stem from auditory deficits. For example, some individuals with ASD have difficulty perceiving prosodic cues to phrase boundaries ( Diehl *et al.*, 2008) and linguistic focus ( Peppé *et al.*, 2011). ASD has also been linked to impaired duration discrimination ( Brenner *et al.*, 2015; Karaminis *et al.*, 2016; Martin *et al.*, 2010) but preserved pitch discrimination and memory for pitch sequences ( Heaton *et al.*, 2008; Jarvinen-Pasley *et al.*, 2008b; Stanutz *et al.*, 2014). If prosodic deficits in ASD stem from abnormalities in auditory processing, then they should reflect the unique auditory processing profile of individuals with ASD, and prosodic impairments should be greater for perception and production of duration-based prosodic cues compared to pitch-based prosodic cues. On the other hand, if impairments are present across all conditions, regardless of the acoustic cue presented, this would suggest that prosodic difficulties in ASD stem primarily from modality-general deficits in the understanding of emotional and pragmatic aspects of language.

### The role of pitch and durational cues in focus and phrase perception

Speech tends to be structurally *degenerate*, i.e. a given speech category is often conveyed by multiple acoustic cues simultaneously. This property may make speech robust to both external background noise ( Winter, 2014) and internal “noise” related to imprecise representation of auditory information ( Patel, 2014). In support of this idea, we found that performance on the Combined cues condition surpassed that of either single-cue condition for both phrase perception and focus perception, in alignment with previous findings that rising pitch and increased duration are more effective cues to phrase boundaries when presented simultaneously ( Cumming, 2010).

### Limitations

The MBOPP currently has several limitations which should be kept in mind by users but could be addressed in future versions of the battery. First, all test items were spoken by a single talker. As a result, the relative usefulness of pitch versus duration cues for a given prosodic feature may reflect that talker’s idiosyncratic patterns of cue use rather than, more generally, the usefulness of those cues across talkers. Second, only English test items are included, specifically, from a speaker of Standard Southern British English. It seems uncontroversial to say that, although spoken by a minority, this accent is widely understood across the English-speaking world, so we expect a high level of familiarity with this accent from TV, films, newscasts and teaching materials, at least. However, it is possible that British residents may have some advantage on this test due to greater familiarity with this accent. We consider the use of SSBE here a starting point, and a worthwhile goal for future research would be to develop additional versions of the battery targeted at speakers of other varieties of English. A third limitation is that, currently, only two aspects of prosody perception are included, focus perception and phrase boundary detection. Stress perception and emotion perception are two particularly important aspects of prosody perception which will be included in future versions.

## Data availability

### Underlying data

Multidimensional Battery of Prosody Perception. OSF: http://doi.org/10.17605/OSF.IO/EAQBJ ( Jasmin, 2021)

MBOPP_Data.csv contains deidentified results for each battery item for each participant.
