# Supplementary material for: The Multidimensional Battery of Prosody Perception (MBOPP)
Source: Wellcome Open Res. 2021 Oct 6;5:4. Originally published 2020 Jan 8. [Version 2] doi: 10.12688/wellcomeopenres.15607.2 (PMC8881696; doi:10.12688/wellcomeopenres.15607.2)
Supplement: Peer review history

# Peer review: Reviewer response for version 2

**DOI:** 10.21956/wellcomeopenres.19044.r46279

**Published:** 2022-02-25

**Authors:** Margriet A. Groen

**Affiliations:** 1 Department of Psychology, Lancaster University, Lancaster, UK

My sincere apologies for the delay in reading the revised manuscript. I have now had a chance to do so and in this thorough and thoughtful revision, the authors address all the suggestions and concerns I articulated in my original review. I have no further comments to make and am happy to approve it for indexing.

Is the rationale for developing the new method (or application) clearly explained?

Yes

Is the description of the method technically sound?

Yes

Are the conclusions about the method and its performance adequately supported by the findings presented in the article?

Yes

If any results are presented, are all the source data underlying the results available to ensure full reproducibility?

Yes

Are sufficient details provided to allow replication of the method development and its use by others?

Yes

Reviewer Expertise:

Associations between phonological processing (incl. segmental/phonemic and suprasegmental/prosodic processing) and reading development. Assessment of segmental and suprasegmental aspects of speech. Language and literacy development more broadly.

I confirm that I have read this submission and believe that I have an appropriate level of expertise to confirm that it is of an acceptable scientific standard.

# Peer review: Reviewer response for version 2

**DOI:** 10.21956/wellcomeopenres.19044.r46280

**Published:** 2021-10-25

**Authors:** Robert Fuchs

**Affiliations:** 1 University of Hamburg, Hamburg, Germany

I'm happy to approve this. I have no further comments.

Is the rationale for developing the new method (or application) clearly explained?

Yes

Is the description of the method technically sound?

Yes

Are the conclusions about the method and its performance adequately supported by the findings presented in the article?

Yes

If any results are presented, are all the source data underlying the results available to ensure full reproducibility?

Partly

Are sufficient details provided to allow replication of the method development and its use by others?

Partly

Reviewer Expertise:

Acoustic phonetics, sociolinguistics, varieties of English, Second Language Acquisition

I confirm that I have read this submission and believe that I have an appropriate level of expertise to confirm that it is of an acceptable scientific standard.

# Peer review: Reviewer response for version 1

**DOI:** 10.21956/wellcomeopenres.17096.r37918

**Published:** 2020-03-23

**Authors:** Robert Fuchs

**Affiliations:** 1 University of Hamburg, Hamburg, Germany

The Multidimensional Battery of Prosody Perception presented in this paper will be extremely useful for researchers in a number of areas. In fact, I might very well use it my own research. I thus wholeheartedly endorse its indexing, provided that a few points mentioned below are addressed. In addition to a few minor comments, more substantial comments relate to the statistical analysis of the data (mixed effect regression modelling should be used in order to control for non-independence of individual trials) and the experimental data being published on the online annex (it appears that average across trials are available, but not data for each trial).

 Data files: The authors should publish the raw data of experiments coming from the experiments so that other researchers can directly conduct statistical tests comparing their results with yours. The file MBOPP-data.csv does not seem to include this data (Even if it did include data from the experimental results, it would seem to include one data point per condition and participant. This does not appear to be the entire dataset, but averaged results.) In principle, the authors should strive to make as much data available as the protection of the anonymity of the participants allows. This includes information on the outcome of every single trial in the experiment, which stimulus was tested (cf. numbers in the tables containing the stimuli), age, gender and other information on the participants. Without this information, other researchers will not be able to conduct statistical comparisons of their data with your data, making the present data much less useful than it could be.

 Dialectal variation: Is the Battery equally suitable for speakers of Southern Standard British English, Manchester English, Scottish English, American English etc.? What is the native accent of the actor who recorded the stimuli? Might participants’ familiarity with the native accent of the actor influence performance on the test?

 p.3, “speech perception is thought to be categorizing” -> reference required

 p.3, “acoustic patterns on slower time scales” -> “acoustic patterns on longer time scales”

 p.3, “Not only has dyslexia has been linked” -> “Not only has dyslexia been linked”

 p.4, “However, because these tests do use actual language, they arguably measure auditory discrimination rather than prosody perception per se.” -> I don’t find this conclusion convincing: Naturalistic stimuli may indeed provide insight into the processing of prosody, if the task is carefully designed.

 p.4, “The most widely used battery of prosody perception available for purchase” -> This implies there are others as well. They should be discussed here, at least briefly.

 p.5, “morphed together to create intermediate versions” -> I have no personal experience with STRAIGHT, but from my experience with other software I understand that creating truly intermediate versions is not possible. What is possible is, given two recordings A and B, to take one of them (say A) and resynthesise it with any durational pattern or any pitch contour, including ones that are intermediate between the durational patterns and pitch contours of A and B. However, the resynthesised version will retain all other voice characteristics of A and thus not be a truly intermediate version of A and B.

 p.7, “The task could be made more difficult” -> “The task could be made yet more difficult”

 p.11, Figure 3 -> Indicate significant differences with asterisks and braces (in this figure and others of the same type)

 p.11, “Cronbach’s alpha was used to calculate reliability” -> Briefly define how reliability is calculated here

 p.12, “Relationship between conditions” -> What is being compared to what here? For focus-focus or phrase-phrase conditions, I assume it is the same trial (i.e. participants and stimulus identical). But what about focus-phrase correlations? Since the sentences vary, there would seem to be a large number of possible conditions to match in the correlations.

 p.13, “by conducting two multiple linear regressions” -> Mixed effects regression models with participant and sentence as random factors would be more appropriate here. Linear regression ignores the non-independence of multiple datapoints here and will lead to an increased risk of spurious results.

 p.13, “as the dependent variable. For focus perception,… in the both cues of condition” -> Beta (ß) is not a good measure of explained variance, as the authors seem to imply. Instead, use measures such as(Pseudo) R2, ROC etc.

 p.13, “This instrument could facilitate investigation of a number of research questions” -> Dialectal variation is another field of application, for example, see the psycholinguistic/sociolinguistic applications in Fuchs, Robert. 2016. Speech Rhythm in Varieties of English. Evidence from Educated Indian English and British English. Singapore: Springer.

 p.14, “In support of this idea, we found that performance on the both cues condition surpassed that of either single-cue condition for phase perception” -> But this is the opposite of redundancy. One cue adds information that the other does not provide, hence in the both cues condition performance is better than in either of the single cue conditions. Instead, redundancy comes into play here in that the two cues are not completely orthogonal, i.e. performance in the both cues condition is not simply the sum of performance in the two single cue conditions (discounting ceiling effects).

Is the rationale for developing the new method (or application) clearly explained?

Yes

Is the description of the method technically sound?

Yes

Are the conclusions about the method and its performance adequately supported by the findings presented in the article?

Yes

If any results are presented, are all the source data underlying the results available to ensure full reproducibility?

Partly

Are sufficient details provided to allow replication of the method development and its use by others?

Partly

Reviewer Expertise:

Acoustic phonetics, sociolinguistics, varieties of English, Second Language Acquisition

I confirm that I have read this submission and believe that I have an appropriate level of expertise to confirm that it is of an acceptable scientific standard, however I have significant reservations, as outlined above.

## References

[1] : Speech Rhythm in Varieties of English.2016; 10.1007/978-3-662-47818-9 10.1007/978-3-662-47818-9

# Response

**Published:** 2021-09-17

**Authors:** Kykle Jasmin

**Affiliations:** Birkbeck University of London, UK

**Robert Fuchs:**

 The Multidimensional Battery of Prosody Perception presented in this paper will be extremely useful for researchers in a number of areas. In fact, I might very well use it my own research. I thus wholeheartedly endorse its indexing, provided that a few points mentioned below are addressed. In addition to a few minor comments, more substantial comments relate to the statistical analysis of the data (mixed effect regression modelling should be used in order to control for non-independence of individual trials) and the experimental data being published on the online annex (it appears that average across trials are available, but not data for each trial).

**Thanks for the kind words about the study – we’re glad you like it. Apologies for the long delay in revision. We agree with your assessment of the statistics, and so we have re-run the tests online such that each participant judged each item in each condition, and re-done the stats using mixed effects models for most cases (excepting the scatterplots of performance correlations between different conditions).**

 Data files: The authors should publish the raw data of experiments coming from the experiments so that other researchers can directly conduct statistical tests comparing their results with yours. The file MBOPP-data.csv does not seem to include this data (Even if it did include data from the experimental results, it would seem to include one data point per condition and participant. This does not appear to be the entire dataset, but averaged results.) In principle, the authors should strive to make as much data available as the protection of the anonymity of the participants allows. This includes information on the outcome of every single trial in the experiment, which stimulus was tested (cf. numbers in the tables containing the stimuli), age, gender and other information on the participants. Without this information, other researchers will not be able to conduct statistical comparisons of their data with your data, making the present data much less useful than it could be.

**We agree, and the new data reflects this change.**

 Dialectal variation: Is the Battery equally suitable for speakers of Southern Standard British English, Manchester English, Scottish English, American English etc.? What is the native accent of the actor who recorded the stimuli? Might participants’ familiarity with the native accent of the actor influence performance on the test?

**This is an interesting question. The speaker was from Reading, England, and his accent is probably best described as Standard Southern British English. It seems uncontroversial to say that, although spoken by a minority, this accent is widely understood across the English-speaking world, so we expect a high level of familiarity with this accent from TV, films, newscasts and teaching materials, at least. It's possible that British residents may have some advantage on this test due to greater familiarity with this accent, but it would be difficult to avoid some limitations along these lines due to the great variety in English accents present worldwide. A worthwhile goal for future research would be to develop additional versions of the battery targeted at speakers of other varieties of English.**

**We now include the following text in the Discussion section on Limitations:**

**“It seems uncontroversial to say that, although spoken by a minority, this accent is widely understood across the English-speaking world, so we expect a high level of familiarity with this accent from TV, films, newscasts and teaching materials, at least. However, it is possible that British residents may have some advantage on this test due to greater familiarity with this accent. We consider the use of SSBE here a starting point, and a worthwhile goal for future research would be to develop additional versions of the battery targeted at speakers of other varieties of English.”**

 p.3, “speech perception is thought to be categorizing” -> reference required

**We now cite:**

**Holt, L. L., & Lotto, A. J. (2010). Speech perception as categorization. *Attention, Perception, & Psychophysics*, *72*(5), 1218-1227.**

 p.3, “acoustic patterns on slower time scales” -> “acoustic patterns on longer time scales”

**Corrected.**

 p.3, “Not only has dyslexia has been linked” -> “Not only has dyslexia been linked”

**Corrected.**

 p.4, “However, because these tests do use actual language, they arguably measure auditory discrimination rather than prosody perception per se.” -> I don’t find this conclusion convincing: Naturalistic stimuli may indeed provide insight into the processing of prosody, if the task is carefully designed.

Apologies – there was a missing “not” in that sentence. The mentioned tests do *not* use actual language.

 p.4, “The most widely used battery of prosody perception available for purchase” -> This implies there are others as well. They should be discussed here, at least briefly.

**Apologies if this is unclear. We do refer to a few other tests in the following paragraph:**

**“** **Moreover, there are a number of examples of ceiling effects in the literature on prosody perception in adolescents and adults in research using other prosody perception tests (Chevallier *et al.*, 2008; Lyons *et al*., 2014; Paul *et al*., 2005), suggesting that existing methodologies for testing prosody perception are insufficiently challenging for adult participants. Research on prosody would be facilitated by a publicly available test with adaptive difficulty suitable for a range of ages and backgrounds.”**

 p.5, “morphed together to create intermediate versions” -> I have no personal experience with STRAIGHT, but from my experience with other software I understand that creating truly intermediate versions is not possible. What is possible is, given two recordings A and B, to take one of them (say A) and resynthesise it with any durational pattern or any pitch contour, including ones that are intermediate between the durational patterns and pitch contours of A and B. However, the resynthesised version will retain all other voice characteristics of A and thus not be a truly intermediate version of A and B.

**Thanks for this. STRAIGHT functions differently from more traditional resynthesis in that both A and B are first decomposed into their power spectrum, fundamental frequency, and an aperiodic component. The power spectrum and aperiodic component are the basis for resynthesizing the other voice characteristics (frequency of sibilants, distribution of formants), all of which are set by default to be intermediate between the two recordings. Because these characteristics are estimated by STRAIGHT for both recordings, it is possible to synthesize ‘naturalistic’ intermediate morphs not just between different tokens from the same talker, but between different talkers with widely varying speech.**

**To clarify this, we have added this to the last paragraph of the Introduction:**

**“Speech morphing software (STRAIGHT, Kawahara & Irino, 2005) was then used to decompose these two recordings, align them onto one another, and resynthesize (“morph”) them such that the extent to which pitch and durational patterns cued one prosodic interpretation or the other could be varied independently *while all other acoustic characteristics are set to be intermediate between the two recordings.*”**

 p.7, “The task could be made more difficult” -> “The task could be made yet more difficult”

**Corrected.**

 p.11, Figure 3 -> Indicate significant differences with asterisks and braces (in this figure and others of the same type)

**We have made this change.**

 p.11, “Cronbach’s alpha was used to calculate reliability” -> Briefly define how reliability is calculated here

**We now describe how alpha was calculated:**

**“Cronbach’s alpha was used to calculate reliability for each of the six subtests by first (for each condition and test) creating a matrix with a row for each subject, a column for each item, and the performance score (1 vs 0) as the value, and then submitting this matrix to the alpha function in R’s *psych* package (Revelle, 2016).”**

 p.12, “Relationship between conditions” -> What is being compared to what here? For focus-focus or phrase-phrase conditions, I assume it is the same trial (i.e. participants and stimulus identical). But what about focus-phrase correlations? Since the sentences vary, there would seem to be a large number of possible conditions to match in the correlations.

**Here we briefly depart from the use of mixed effects models to simply report the proportion correct (performance) on each subject, correlated with performance on each other sub-test. We have amended the text to make this clearer:**

**“Pearson’s correlations were used to examine the relationship between performance *(proportion correct response for each subject)* across all six subtests.”**

 p.13, “by conducting two multiple linear regressions” -> Mixed effects regression models with participant and sentence as random factors would be more appropriate here. Linear regression ignores the non-independence of multiple datapoints here and will lead to an increased risk of spurious results.

**We now report this using linear mixed effects models.**

 p.13, “as the dependent variable. For focus perception,… in the both cues of condition” -> Beta (ß) is not a good measure of explained variance, as the authors seem to imply. Instead, use measures such as(Pseudo) R2, ROC etc.

**As an effect size measure, we now report odds ratios and Z scores for the terms in the mixed effects logistic regressions.**

 p.13, “This instrument could facilitate investigation of a number of research questions” -> Dialectal variation is another field of application, for example, see the psycholinguistic/sociolinguistic applications in Fuchs, Robert. 2016. Speech Rhythm in Varieties of English. Evidence from Educated Indian English and British English. Singapore: Springer.

**We now mention this research avenue in the first paragraph of the discussion**

**“Another avenue of investigation would be dialectal variation (see Fuchs, 2016), e.g. whether speakers of other varieties of English are able to use pitch and duration similarly. Second language learning may also be a fruitful line of research using the battery. Indeed, we have recently shown that L2 English speakers of L1 Mandarin tend to perceptually weight pitch highly in perception of English speech (Jasmin *et al.*, 2021).”**

 p.14, “In support of this idea, we found that performance on the both cues condition surpassed that of either single-cue condition for phase perception” -> But this is the opposite of redundancy. One cue adds information that the other does not provide, hence in the both cues condition performance is better than in either of the single cue conditions. Instead, redundancy comes into play here in that the two cues are not completely orthogonal, i.e. performance in the both cues condition is not simply the sum of performance in the two single cue conditions (discounting ceiling effects).

**Thank you for the thoughtful critique. We believe a change in terminology is necessary here – namely that multiple cues indexing the same feature to ensure robustness is referred to as ‘degeneracy’ in biology and more recently in language science (Winter, 2014). We have amended the paragraph as follows:**

**“Speech tends to be structurally *degenerate*, i.e. a given speech category is often conveyed by multiple acoustic cues simultaneously. This property may make speech robust to both external background noise ( Winter, 2014) and internal “noise” related to imprecise representation of auditory information ( Patel, 2014). In support of this idea, we found that performance on the Combined cues condition surpassed that of either single-cue condition for both phrase perception and focus perception, in alignment with previous findings that rising pitch and increased duration are more effective cues to phrase boundaries when presented simultaneously ( Cumming, 2010).”**

# Peer review: Reviewer response for version 1

**DOI:** 10.21956/wellcomeopenres.17096.r37746

**Published:** 2020-03-03

**Authors:** Margriet A. Groen

**Affiliations:** 1 Department of Psychology, Lancaster University, Lancaster, UK

Dear Dr. Jasmin and colleagues,

 I've enjoyed reading this well-written manuscript, describing what I believe to be an innovative and relevant new measure of two aspects of prosody perception (focus perception and phrase boundary perception).

 You clearly describe the rationale for its development and set-up. I find the use of morphing software to create the stimuli particularly relevant as it allows tighter experimental control over: 1) the degree to which particular cues are present in the stimulus; and 2) over item difficulty.

 I do have some suggestions that I believe would improve the manuscript.

 In the introduction, under 'Prosody and reading acquisition', you discuss work linking perception of prosody to word reading, but you don't mention work on the relationship between prosodic processing and reading comprehension. There is a substantial literature on this and some of it you refer to in the manuscript (e.g., Whalley & Hansen, 2006; Lochrin et al., 2015) but only in the context of word reading. It would be relevant to point to the relation to reading comprehension as well. Holliman et al. (2014) ^1^ is another relevant paper. Additionally, some of my own work suggests that children with poor reading comprehension have deficits in prosodic processing, and in particular in speech rhythm perception. You might also want to refer to the 'implicit prosody hypothesis' (Fodor, 1998) ^2^ in this context. Also relevant is Kentner (2012) ^3^.

 In the methods section, you refer to the three conditions as 'Pitch-Only', 'Time-Only' and 'Combined'. In the results section (and the figures), however, you refer to 'pitch', 'time' and 'both'. It would be helpful to be consistent throughout the manuscript in the labelling of the conditions.

 In the results section, you report two multiple linear regressions to address the question of whether pitch and time account for unique variance in prosody perception. You use the 'Time-Only' and 'Pitch-Only' conditions as predictors of performance in the 'Combined' condition. I'm not a statistician, but I feel this does not take into account the dependencies in the data, i.e., that the stimulus materials are highly similar across conditions. Responses to the 'Time-Only' version of a sentence are therefore likely to be related to (i.e., NOT independent from) responses to the 'Pitch-Only' version of the same sentence. This increases the chance of Type-I errors. The considerable correlations (between .6 and .9) you report indicate this as well. In my view, it would be more appropriate to fit mixed-effects models to the data in which you specify a random effect structure that accounts for the item-dependencies (as well as the participant-dependencies). Lazic (2010) ^4^ and Winter (2011) ^5^ explain the problem of dependencies in more detail. Winter's new book 'Statistics for Linguists: An Introduction using R' provides a highly intuitive introduction to this problem and its solution (mixed effects models). As yours is primarily a methods paper, I have not listed this as a major revision. I nevertheless feel it would be important to do, or at least provide item-level data (i.e., all responses to all items for all participants), which would allow others to do it.

 In the data-file, there are three columns that do not seem to be mentioned in the manuscript (prosody_both, prosody_pitch, prosody_time). It would be helpful to clarify what they refer to.

Is the rationale for developing the new method (or application) clearly explained?

Yes

Is the description of the method technically sound?

Yes

Are the conclusions about the method and its performance adequately supported by the findings presented in the article?

Yes

If any results are presented, are all the source data underlying the results available to ensure full reproducibility?

Yes

Are sufficient details provided to allow replication of the method development and its use by others?

Yes

Reviewer Expertise:

Associations between phonological processing (incl. segmental/phonemic and suprasegmental/prosodic processing) and reading development. Assessment of segmental and suprasegmental aspects of speech. Language and literacy development more broadly.

I confirm that I have read this submission and believe that I have an appropriate level of expertise to confirm that it is of an acceptable scientific standard, however I have significant reservations, as outlined above.

## References

[1] : Beginning to disentangle the prosody-literacy relationship: a multi-component measure of prosodic sensitivity. *Reading and Writing* .2014;27(2) : 10.1007/s11145-013-9443-6 255-266 10.1007/s11145-013-9443-6

[2] : *Journal of Psycholinguistic Research* .1998;27(2) : 10.1023/A:1023258301588 285-319 10.1023/A:1023258301588

[3] : Linguistic rhythm guides parsing decisions in written sentence comprehension. *Cognition* .2012;123(1) : 10.1016/j.cognition.2011.11.012 1-20 10.1016/j.cognition.2011.11.012 22217385

[4] : The problem of pseudoreplication in neuroscientific studies: is it affecting your analysis?. *BMC Neurosci* .2010;11: 10.1186/1471-2202-11-5 5 10.1186/1471-2202-11-5 20074371PMC2817684

[5] : Pseudoreplication in Phonetic Research. *ICPhS XVII* .2011;

[6] : The role of prosody in reading comprehension: evidence from poor comprehenders. *Journal of Research in Reading* .2019;42(1) : 10.1111/1467-9817.12133 37-57 10.1111/1467-9817.12133

# Response

**Published:** 2021-09-17

**Authors:** Kykle Jasmin

**Affiliations:** Birkbeck University of London, UK

**Margariet Groen:**

 Dear Dr. Jasmin and colleagues,

 I've enjoyed reading this well-written manuscript, describing what I believe to be an innovative and relevant new measure of two aspects of prosody perception (focus perception and phrase boundary perception).

**Thank you for these kind words. We’re glad you liked the paper. Apologies for the long turnaround on this revision. We needed to collect new data to address your suggestions.**

 You clearly describe the rationale for its development and set-up. I find the use of morphing software to create the stimuli particularly relevant as it allows tighter experimental control over: 1) the degree to which particular cues are present in the stimulus; and 2) over item difficulty.

 I do have some suggestions that I believe would improve the manuscript.

 In the introduction, under 'Prosody and reading acquisition', you discuss work linking perception of prosody to word reading, but you don't mention work on the relationship between prosodic processing and reading comprehension. There is a substantial literature on this and some of it you refer to in the manuscript (e.g., Whalley & Hansen, 2006; Lochrin et al., 2015) but only in the context of word reading. It would be relevant to point to the relation to reading comprehension as well. Holliman et al. (2014) ^1^ is another relevant paper. Additionally, some of my own work suggests that children with poor reading comprehension have deficits in prosodic processing, and in particular in speech rhythm perception. You might also want to refer to the 'implicit prosody hypothesis' (Fodor, 1998) ^2^ in this context. Also relevant is Kentner (2012) ^3^ .

**Thank you for the suggestion. We have expanded the introduction so that it now includes a review of research linking prosodic processing to reading comprehension, including the references you suggest here:**

**“The link between prosody and reading is not limited to word reading, as prosody perception and production have also been shown to be related to reading comprehension (Holliman et al., 2014). Prosody predicts reading comprehension even when a variety of additional linguistic variables are accounted for, including phonological skills and vocabulary (Whalley & Hansen, 2006; Holliman et al., 2010b; Lochrin et al., 2015; Breen et al., 2016), syntactic awareness (Veenendaal et al., 2014), and decoding (Groen et al., 2019). This link between prosodic skills and reading comprehension could reflect links between prosodic and syntactic processing during reading. Fodor (1998), for example, proposed that readers generate prosodic contours during silent reading, and that these prosodic structures can affect syntactic parsing decisions, a hypothesis later supported by eye-tracking data (Kentner, 2012).”**

 In the methods section, you refer to the three conditions as 'Pitch-Only', 'Time-Only' and 'Combined'. In the results section (and the figures), however, you refer to 'pitch', 'time' and 'both'. It would be helpful to be consistent throughout the manuscript in the labelling of the conditions.

**We now consistently use the simpler terms “Pitch”, “Duration”, and “Combined”.**

 In the results section, you report two multiple linear regressions to address the question of whether pitch and time account for unique variance in prosody perception. You use the 'Time-Only' and 'Pitch-Only' conditions as predictors of performance in the 'Combined' condition. I'm not a statistician, but I feel this does not take into account the dependencies in the data, i.e., that the stimulus materials are highly similar across conditions. Responses to the 'Time-Only' version of a sentence are therefore likely to be related to (i.e., NOT independent from) responses to the 'Pitch-Only' version of the same sentence. This increases the chance of Type-I errors. The considerable correlations (between .6 and .9) you report indicate this as well. In my view, it would be more appropriate to fit mixed-effects models to the data in which you specify a random effect structure that accounts for the item-dependencies (as well as the participant-dependencies). Lazic (2010) ^4^ and Winter (2011) ^5^ explain the problem of dependencies in more detail. Winter's new book 'Statistics for Linguists: An Introduction using R' provides a highly intuitive introduction to this problem and its solution (mixed effects models). As yours is primarily a methods paper, I have not listed this as a major revision. I nevertheless feel it would be important to do, or at least provide item-level data (i.e., all responses to all items for all participants), which would allow others to do it

**Thank you for the suggestion; new data has been collected such that each participant saw each item in each condition, and stats have been re-run using mixed effects models to account for these item-wise dependencies. Throughout the paper we now use mixed effects models, with the exception of when examining correlations between performance across all 6 sub-tests. However, we also now publish the complete trial-wise dataset so readers can reanalyse the data as they prefer and as methods develop.**

 In the data-file, there are three columns that do not seem to be mentioned in the manuscript (prosody_both, prosody_pitch, prosody_time). It would be helpful to clarify what they refer to.

**These columns are not present in the new data file.**
